# Induced pluripotent stem cell: A headway in reprogramming with promising approach in regenerative biology

**DOI:** 10.14202/vetworld.2017.640-649

**Published:** 2017-06-14

**Authors:** N. Rawat, M. K. Singh

**Affiliations:** Embryo Biotechnology Lab, Animal Biotechnology Centre, ICAR - National Dairy Research Institute, Karnal - 132 001, Haryana, India

**Keywords:** cellular reprogramming, embryonic stem cells, induced pluripotent stem cells, stem cells

## Abstract

Since the embryonic stem cells have knocked the doorsteps, they have proved themselves in the field of science, research, and medicines, but the hovered restrictions confine their application in human welfare. Alternate approaches used to reprogram the cells to the pluripotent state were not up to par, but the innovation of induced pluripotent stem cells (iPSCs) paved a new hope for the researchers. Soon after the discovery, iPSCs technology is undergoing renaissance day by day, i.e., from the use of genetic material to recombinant proteins and now only chemicals are employed to convert somatic cells to iPSCs. Thus, this technique is moving straightforward and productive at an astonishing pace. Here, we provide a brief introduction to iPSCs, the mechanism and methods for their generation, their prevailing and prospective applications and the future opportunities that can be expected from them.

## Introduction

Stem cells are the special primal cells which retain two peculiar properties: Self-renewal to persist their undifferentiated state and differentiation potential to give rise to specialized cell types. On the basis of differentiation ability, they are categorized as totipotent, pluripotent, and multipotent stem cells. On the basis of origin, they can be categorized as embryonic stem cells (ESCs), derived from inner cell mass cells of blastocysts, and can differentiate into all the derivatives of three primary germ layers. The other cell types are adult stem cells, present in adult tissues. These are multipotent cells which are lineage specific and produce a limited number of cell types [1]. The ESCs possess all the qualities required for their contribution in scientific research and medicinal purposes, but they have limitations as immunologic rejection, and raising ethical and moral concerns because generation of ESCs requires the destruction of embryos [2]. To overcome these drawbacks, attempts have been taken to achieve individual-specific pluripotent stem cells by somatic cell nuclear transfer [3], treatment with an extract of pluripotent cells [4] and cell fusion [5]. All these approaches of nuclear reprogramming are accompanied by frailties as a technical inconvenience, partial reprogramming, and tetraploid formation, respectively, which hamper their use in medical science and technology [6].

To address these shortcomings, Takahashi and Yamanaka in 2006 [6] introduced an advanced step toward nuclear reprogramming based on transcription factor action for generation of new stem cell type called “induced pluripotent stem cells (iPSCs).” They tried 24 candidate factors for their ability to induce pluripotency and concluded that four fundamental transcription factors Oct-3/4, Sox2, c-Myc, and Klf4 (also called Yamanaka factors) are vital for conversion of mouse fibroblasts into pluripotent stem cells. This innovative discovery has changed the view about development and cellular specialization and hence rewarded by the Nobel Prize in 2012.

## Comparison between iPS and ES Cells

iPSCs share many similarities with ESCs in terms of their morphology, surface markers expression, feeder dependence, and *in vivo* teratoma formation capacity. The *in vitro* differentiation potential of iPSCs is distinctive as compared to ESCs. Furthermore, the DNA methylation pattern between iPSCs and ESCs has revealed certain differentially methylated regions. Thus, the epigenetic status of iPSCs partially differs from ESCs which may be due to the epigenetic memory of the cell type from where it is originated [7] (Table-1).

**Table-1:** Comparison between iPSCs and ESCs.

Features	iPSCs	ESCs
Source of generation	Somatic cells	Embryos
Ease of generation	Technically straightforward	Expertise required
Nature of cells	Pluripotent	Pluripotent
Teratoma formation	Yes	Yes
Self-renewal capacity	Yes	Yes
Pre-mature aging	Yes	No
Telomerase activity	High	High
Immuno-rejection	No	Yes
Abnormalities in cells	High	Low
Clinical applications	Widely used	Restricted
Ethical concerns	No	Yes

iPSCs=Induced pluripotent stem cells, ESCs=Embryonic stem cells

The iPSCs and ESCs are categorized into two states-naive and prime; distinguished by morphology, gene expression pattern, and external signal dependence. Naive state cells are conventionally mouse-type ES/iPSCs, which form compact dome-shaped colonies and proliferate very rapidly. They are dependent on external LIF signals for their growth and proliferation. On the contrary, prime state cells are human type ES/iPSCs, which form flat colonies and dependent on the basic fibroblast growth factor (bFGF) signal for their proliferation [8]. They proliferate very slowly as compared to naive state cells. They are also called epiblast stem cells (EpiSCs). The EpiSCs have the potential to differentiate into various cell types but could not form chimeric mice when injected into blastocyst. This represents the difference of their developmental stage in comparison to naive state cells. This developmental hurdle can be recovered by the introduction of pluripotent genes which can convert their status to mouse ES-like cells [9].

## Mechanism of Path Switching

### How the process starts

The first noticeable change that takes place during the reprogramming of fibroblasts is mesenchymal-epithelial transition (MET), reversal of epithelial-mesenchymal transition (EMT), which occurs during early gastrulation. The two main genes involved in switching of EMT and MET states are E-cadherin and Snail. Upregulation of E-cadherin is crucial for initiating MET process, and upregulation of Snail is responsible for gene reorganization during EMT. E-cadherin is a transmembrane constituent of intercellular adherens junctions responsible for maintaining epithelial cohesion. The snail is a basic helix-loop-helix transcription factor that binds to specific sequences called E-boxes and represses the transcription of E-cadherin and other key epithelial regulators [10]. Thus, the exogenous reprogramming factors inhibit the key mesenchymal genes and trigger an epithelial program to overcome the EMT epigenetic barrier of fibroblasts for inducing the state of pluripotency in them.

### The regulatory pathways involved

**Figure-1:**
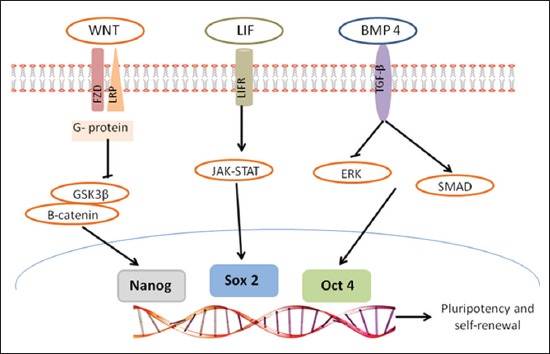
Signaling pathways and transcription factors regulates cell stemness.

The extrinsic stimuli and intrinsic circuitries play a synergistic role in sustaining the undifferentiated and self-renewing state of cells. The influential factors involved in maintaining pluripotency are leukemia inhibitory factor (LIF), transforming growth factors (TGFs), and FGFs. LIF is a member of the interleukin-6 cytokines. It is a key factor in maintaining pluripotency by inhibiting differentiation. On LIF binding to the receptor, JAK-STAT pathways becomes activated. STAT3 self phosphorylates themselves to forms a homodimer, which translocates to the nucleus, where it binds to gene enhancers and regulates target gene expression. Thus, the LIF/JAK/STAT3 pathway maintains pluripotency [11] (Figure-1).

Another pathway involved in the maintenance of pluripotency is TGF-β superfamily which includes TGF-β, activin, inhibin, growth differentiation factor, bone morphogenetic proteins, etc. The TGF-β members follow signaling pathway by binding to heteromeric complexes of serine/threonine kinase receptors, which subsequently activate intracellular Smad proteins. Thus, TGF-β-related signaling pathways are crucially involved in regulating the pluripotency and cell fate of ESCs [12]. FGF signaling also plays a decisive role in maintaining ESCs pluripotency. In consistence, withdrawal of FGFs or interference of FGF signaling by FGF receptor (FGFR) inhibitors causes cellular differentiation. FGFs signaling begins by binding to FGFRs which further triggers diverse signaling cascades, including MAPKs pathway, JAK/STAT pathway, PI3K pathway, and PLC pathway. Furthermore, FGF2 helps in maintaining the cells in their undifferentiated state by inducing the feeder layer cells to secrete TGF-β1 and insulin-like growth factor 2 [13].

The primary role of the reprogramming factors at the early phase is to reverse the EMT process and to initiate MET. TGF-β signaling pathway is the primary target of the reprogramming factors: Tgf β1 and Tgf β3, two members of TGF-β superfamily, are repressed by c-Myc and Oct4/Klf4, respectively, Tgf βR2 is suppressed by c-Myc, Tgf βR3 is suppressed by Oct4/Sox2. The silencing of TGF-β signaling leads to the inhibition of expression of Snail, which is critical for EMT. Klf4 activates E-cadherin and other epithelial program to start MET. Thus, there is the peculiar distribution of task among the transcription factors for reprogramming the fibroblast cells to pluripotent state [14,15].

## Methods of Generation of iPSCs

Direct reprogramming of somatic cells by introducing a set of known genes to revert them to embryonic state is a very promising approach. This technique involves retroviral and lentiviral vectors with strong promoters for higher efficiency which integrate their genome into the host chromosome. The other class of vectors are non-integrating type which includes viral as well as non-viral vectors. Alternative method to generate iPSCs is substitution of genetic factors with small molecules which alters the regulatory pathways and epigenetic state of the cells.

### Integrating method

The pioneers of this technique, Yamanaka and Takahashi had used retroviral-mediated transduction of four set of genes (Oct4, Sox2, c-Myc, and Klf4) to re-programme mouse fibroblasts and induces them to ESC like state [16]. Therefore, majority of studies have used integrating types of vector for delivery of transcription factors as they are powerful tools to introduce foreign genes into host chromosomes and hence results in higher efficiency (Table-2) [16-26]. However, they are followed by many challenges for their use in therapeutic purposes. The primary drawback of genome-integrating vectors is potential risks of random and permanent integration of virus into the host genome at multiple sites which can result in insertional mutations and even in tumorigenesis. Thus, efficiency and safety are the two major issues involved in clinical applications of iPSCs. Recently, several approaches have been developed to generate safer transgene- or integration-free iPSCs.

**Table-2:** Generation of iPSCs using retroviral vectors.

Species	Cell type	Genes introduced	References
Mouse	Embryonic fibroblasts	Oct4, Sox2, Nanog, Klf4, c-Myc	[16]
Mouse	Embryonic and adult fibroblasts	Oct4, Sox2, Klf4, c-Myc	[17,18]
Mouse	Cochlear cells	Oct4, Sox2, Klf4	[19]
Canine	Adult fibroblast	Oct4, Sox2, Klf4, c-Myc	[20]
Porcine	Embryonic fibroblast	Oct4, Sox2, Klf4, c-Myc	[21,22]
Bovine	Embryonic fibroblasts	Oct4, Sox2, Nanog, Klf4, c-Myc, Lin28	[23]
Buffalo	Fetal fibroblasts	Oct4, Sox2, Klf4, c-Myc	[24]
Human	Adult fibroblasts	Oct4, Sox2, Klf4, c-Myc	[25,26]

iPSCs=Induced pluripotent stem cells

Lentiviral vectors are another type of integrating vectors which can infect cells that are not actively dividing, unlike retroviral vectors which can infect only dividing cells to efficiently produce iPSCs (Table-3) [27-31].

**Table-3:** Generation of iPSCs using lentiviral vectors.

Species	Cell type	Genes transduced	References
Rat	Adult fibroblasts	Oct4, Sox2, Klf4, c-Myc	[27]
Rabbit	Adult somatic cells	Oct4, Sox2, Klf4, c-Myc	[28]
Goat	Ear fibroblasts	Oct4, Sox2, Klf4, c-Myc, nanog, Lin28, SV40 large T antigen, hTERT	[29]
Bovine	Fetal fibroblast	Oct4, Sox2, Klf4, c-Myc	[30]
Human	Umbilical cord blood mononuclear cells	Oct4, Sox2, Klf4, c-Myc	[31]

iPSCs=Induced pluripotent stem cells

### Non-integrating methods

For generating transgene-free or integration-free iPSCs, a series of non-integrating vectors have been employed which includes viral as well as non-viral vectors. Some of them are discussed below.

#### Viral methods

Adenoviral transduction

Adenoviruses are among the most commonly used vectors next to retroviruses. The virus undergoes receptor-mediated endocytosis, then released in the cytosol and reaches the nucleus for carrying out viral replication and transcription. The translation of the transcripted mRNA occurs within 18 h after infection, and hence, the transgene is expressed with the maximum level at 48-72 h post transduction [32,33]. They manifested high transduction efficiency for cells in quiescent as well as dividing phase and shows high levels of short-term expression.

Sendai virus transduction

Unlike other RNA viruses, Sendai virus escapes the route to the nucleus and replicates in the cytoplasm of infected cells. Thus, it cannot integrate into the host genome and allows expression of transgenes without risk of modification of host genome. Hence, the iPSCs generated are genetically intact and carries the same genome DNA as the original cells [34]. In addition, Sendai virus vector is capable to infect a vast host range and are non-pathogenic to humans. Among viral vectors, it has been considered a quintessential tool for cell reprogramming and stem cell research [35].

#### Non-viral methods

DNA vectors are more stable than viruses and can be conveniently thawed and re-frozen through several usages. The non-viral vectors recently developed for reprogramming are.

Episomal plasmid

Episomal plasmids are based on the Epstein-Barr nuclear antigen-1 (oriP/EBNA-1). They anchor themselves to the host chromatin and replicates in synchrony with the host genome [36,37]. The episomal plasmids are naturally lost at up to 5% per cell division cycle. Thus, they can be utilized for a broader range of applications, including pre-clinical research, and human gene therapy [38].

Transposons

Two main transposable elements use in reprogramming are sleeping beauty and PiggyBac transposon [39]. Sleeping beauty originates from salmonides, where it remains as an inactive element and reawaken by an *in vitro* mutagenesis approach. Piggybac was identified as an active element in the moth *Trichoplusia*. Both elements show transposition activity in mammalian cells without cellular co-factors, but require DNA bending protein HMGB1 and cell cycle regulator Miz1 for their functioning [40]. Both transposon systems have been shown to be suitable for the derivation of iPSCs in different cell lines.

DNA-free delivery

Reprogramming of cells can also achieved by employing recombinant protein rather than using genetic material directly to cells. These protein-based strategies have been successfully demonstrated but are much more complicated to perform as the generation and purification of protein in required quantity is a challenging task [41,42]. Reports on the generation of iPSCs through non-integrating methods are enlisted in Table-4 [32-46].

**Table-4:** Generation of iPSCs through non-integrating methods.

Delivery vehicle	Species	Cell type	Genes involved	References
Adenoviral vector	Mice	Tail tip fibroblasts	c-Myc, Klf4, Oct4, and Sox2	[32]
Adenoviral vector	Human	Fibroblasts	Ascl1, Brn3b and Ngn2	[33]
Sendai virus	Chimpanzee	Blood cells	Oct4, Klf4, Sox2, c-Myc	[34]
Sendai virus	Human	Peripheral-T cells	Oct4, Sox2, Klf4, c-Myc	[35]
Episomal plasmid	Human	Urine derived cells	Oct4, Sox2, Klf4, SV40LT, Lin28, c-Myc	[36]
Episomal plasmid	Human	Blood	Oct4, Sox2, Klf4, c-Myc	[37]
Episomal plasmid	Human	Leukocytes	Oct4, Sox2, Klf4 and SV40T	[38]
Piggybac transposon	Murine	Embryonic fibroblasts	Oct4, Sox2, Klf4, Lin28, c-Myc, Nanog	[39]
Piggybac transposon	Bat	Embryonic fibroblasts	Oct4, Klf4, Sox2, c-Myc, NR5A2, Nanog, Lin28, and bat-specific miR302/367	[40]
Synthetic mRNA	Human and rat	Adult adipose-tissue	Oct4, Sox2, Klf4, and c-Myc	[41]
Synthetic mRNA	Human	Fibroblasts	Oct4, Sox2, Klf4, and c-Myc	[42]
Synthetic mRNA	Human	Fibroblasts	Oct4, Sox2, Klf4, and c-Myc and Glis1	[43]
Recombinant proteins	Mice	Embryonic fibroblasts	Oct4, Sox2, Klf4, and c-Myc	[44]
Recombinant proteins	Human	Fibroblasts	Sox2, Nanog, Klf4 and NR5A2	[45]
Recombinant proteins	Human	Fibroblast	Oct4, Sox2, Klf4, and c-Myc	[46]

iPSCs=Induced pluripotent stem cells

### Culture Conditions for iPSCs

iPSCs have been established in ES cell media with LIF and bFGF as important factors for maintaining pluripotency [47]. O_2_ tension is also an influential aspect for stem cell maintenance and differentiation. Low O_2_ tension (5% O_2_) condition, called hypoxia, promotes the reprogramming adequacy in both mouse and human fibroblasts [48]. Moreover, to boost the productivity of iPSCs and lessen the burden of exogenous factors, some natural and synthetic small molecules have been proved to be boon. These small molecules target signal transduction pathways that are involved in stem cell renewal and differentiation as Wnt pathway, tyrosine kinase receptor pathway affects epigenetic status of cells by modulating enzymes such as histone deacetylase, histone demethylase, histone methyltransferase, and DNA methyltransferase; DNA replication and other vital functions. Chemical compounds knock off some limitations of transcription factors based reprogramming approach and offer advantages as they are easily accessible to the cells, cost-effective and non-immunogenic [36,49]. A number of compounds have been determined that can take over the function of reprogramming transcription factors and thus, these chemicals have introduced a new window for generating clinical-grade iPSCs [50,51]. The different categories of compounds which are frequently employed in the fruitful generation of chemically iPSCs are mentioned in Table-5 [52-60].

**Table-5:** Generation of iPSCs using chemical compounds.

Modulating pathway	Chemical	Mechanism/target	References
TGF-ß	RepSox (E-616452)	ALK5 inhibitor	[52]
	SB431542	ALK4, ALK5, ALK7 Inhibitor	[53]
MAPK	PD0325901	MEK1, MEK2 inhibitor	[54,55]
PKA	Forskolin	PKA agonist	[50]
Wnt	CHIR99021	GSK3a, GSK3 ß inhibitor	[54,55]
Nuclear receptor	TTNPB	Binds to retinoic acid receptor	[50]
Histone methylation	BIX 01294	Histone lysine methytransferase inhibitor	[56]
	DZNep	Lysine methyltransferase EZH2 inhibitor	[50]
	Tranylcypromine	Lysine specific demethylase 1 inhibitor	[54]
Histone deacetylation	SAHA	Histone deacetylase inhibitor	[57]
	Sodium butyrate	Histone deacetylase inhibitor	[58]
	Trichostatin A	Histone deacetylase inhibitor	[58]
	Valproic acid	Histone deacetylase inhibitor	[59]
Glycolysis	2,4-dinitrophenol	Oxidative phospholyration uncoupler	[60]
	Fructose-2,6-bisphosphate	Phosphofructokinase 1 activator	[60]

iPSCs=Induced pluripotent stem cells

### Application of iPSCs

iPSCs possess the properties of differentiation and germline transmission, therefore have emerged as a substitute to stem cells in the field of biomedical sciences and research. Since iPSCs can bypass the ethical concerns pertained to ESC derivation and peril of allogeneic rejection; therefore, they serve as potential tools for clinical applications and medical research. Till date, iPSCs have accomplished practical applications in areas such as basic stem cell research, diseases modeling, regenerative medicine, drug discovery, and infertility treatment. iPSCs also have considerable applications in the field of animal research like the generation of transgenic animals, improving cloning efficiency, wildlife conservation, etc.

### Application in basic sciences

The emergence of reprogramming techniques has resolved the relationship between transcription factors, signaling pathways, epigenetics, and transition states of the cell toward pluripotency. The MET and EMT transitions that a cell undergoes while reprogramming is a joint venture of action of core set of transcription factors which modulate the epigenetic state of cells and leads to an alteration in their gene expression. This study of developmental biology provides a roadmap for the process of embryogenesis, gastrulation and differentiation to other tissue types.

Also in the field of cancer research, reprogramming techniques are emerging as one of the most versatile tools to study the mechanistic modeling of human tumorigenesis. The process of reprogramming, pluripotency, lineage specification, and oncogenic transformations are fundamentally related to each other regarding the involvement of cancer-related genes [61,62], pluripotency-associated genes and epigenetic related genes [63]. Many reports are available on iPSC lines derived from variety of human cancers, such as melanoma [64,65], prostate cancer [64], gastrointestinal cancers [66], chronic myeloid leukemia [67], lung cancer [68], breast cancer [69], glioblastoma [70], and sarcomas [71]. These patient-derived iPSCs can provide a better understanding of the niche for cancer progression and building a more relevant disease model. The iPSCs derived from banked cord blood from newborns have been employed to deduce the developmental and molecular mechanisms elementary for sequential progression of cancer from the precancerous cell [72]. Thus, iPSCs generated from cancerous cells can be used as model cell line to understand the elemental molecular mechanisms trailing for cancer initiation and progression and to overcome them.

### Cell transplantation/replacement therapy

The applicability of iPSCs in regenerative medicine was demonstrated in 2007 by Hanna *et al.*, where iPSCs were used to cure sickle cell anemia, a disease caused by single gene defect [73]. The disease-causing mutation was rectified by homologous recombination in iPSCs generated from mouse model. The repaired iPSCs then differentiated into blood-forming progenitor cells which were transplanted into anemic mice and disease was cured.

**Figure-2:**
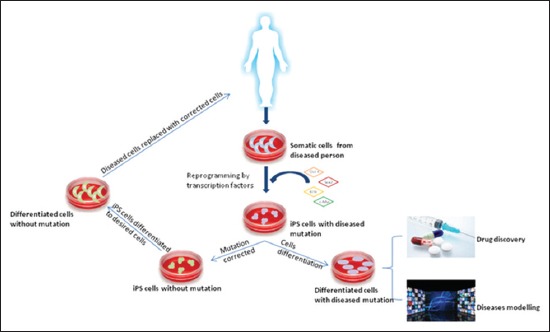
Application of induced pluripotent stem technology in medical science.

Since then many disease have been treated by iPSCs transplantation such as spinal cord injury [74], Parkinson’s disease [75,76], hemophilia A [77], limb ischemia [78], acute myocardial infarction [79,80], peripheral vascular disease [81], diabetes [82], and regeneration [83]. In 2013, a group of scientists at the RIKEN Center for Developmental Biology launched the world’s first clinical trial for the treatment of age-related macular degeneration which results in blindness due to the loss of retinal pigment epithelium. Scientists took skin cells from patients, converted them to iPSCs and then differentiated to retinal pigment epithelium cells. These cells were grown into thin sheets that can be transplanted to the damaged retina [84] (Figure-2).

### Disease modeling

Patient-specific iPSCs serve as “a-patient-in-a-dish” and can be differentiated to any type of body cell which is otherwise difficult to obtain such as brain cells and helps to understand how the disease works. Thus, they have resolved the problem regarding the availability of experimental material. Till date, various tissue specific iPSC derivatives have been generated such as hematopoietic [85,86], hepatic [87,88], endothelial [89], neurological [90-93], and cardiovascular cell lines [71,94,95]. Currently, iPSCs are used as disease models in research of a fatal neuromuscular disorder, spinal muscular atrophy (SMA). SMA patients have a defective SMN1 gene which results in loss of the motor neurons in the spinal cord, leading to muscle wasting, mobility impairment, and early death in children. Somatic cells from the patients suffering from SMA have been reprogrammed to generate SMA iPSCs which have the defective SMN1 gene and are being used as a model to study the onset of the disease in newly developing nerve cells. Furthermore, these SMA iPSCs are used to test a variety of new treatments instead of carrying out them directly on patients [91,96]. In addition, the lab-grown cells help in detecting the symptoms on the onset of diseases which are otherwise difficult to detect. Due to their practical application and successful results more and more diseases are modeled through iPSCs. Patients-specific iPS cell lines have been established for SMA [91], amyotrophic lateral sclerosis [90], familial hypertrophic cardiomyopathy (HCM) [97], β-thalassemia [98], Parkinson’s disease [99,100], Pompe disease [101], Rett syndrome [102], Lesch-Nyhan syndrome [103], LEOPARD syndrome [104], aldehyde dehydrogenase 2 genetic polymorphism [105,106], arrhythmogenic right ventricular dysplasia [107,108], Timothy syndrome [95], long QT syndrome [53,109,110], familial dilated cardiomyopathy [111], and viral cardiomyopathy [112].

### Drug screening and discovery

A plausible assistance is provided by iPSC technology in the creation of a library of animal cell lines that comprises all the major genetic and epigenetic variants of a species. Cell lines derived from these iPSCs represents a realistic and more ideal drug model by creating a “disease-in-a-dish” model over hitherto employed patient’s samples or immortalized tumor-derived cell lines. These well-defined, simulated models help in high-throughput screening of chemical compounds to identify relevant targets. The adoption of iPSCs dependent chemical screening has significantly reformed the success rate of drug development process [96]. After the primary screening, candidate targets are verified for secondary validation screening which holds their pharmacokinetic and safety properties [113]. Primary drug screening has been done for diseases, such as LEOPARD syndrome [104,114], SMA [91], familial dysautonomia [115], diabetic cardiomyopathy [116], neurological disease [117], amyotrophic lateral sclerosis [118], Familial transthyretin amyloidosis, Niemann-Pick disease type C, α1-antitrypsin deficiency, Wilson’s disease [119], and umpteen diseases are under process.

### Treating infertility

Pluripotent stem cells had opened a contemporary perspective in the treatment of patients with azoospermia. Using iPS technology, it is possible to get offspring with the same genetic material by derivation of functional male gametes from the infertile males themselves. Recently, several studies have reported that both mouse iPSCs [120-122] and human iPSCs can differentiate into male germ cells [123,124]. It has been documented that mouse iPSCs can be differentiated to functional spermatozoa [125,126]. Experiments have been conducted to prove that spermatozoa derived from iPSCs are successful in fertilizing the oocytes after intracytoplasmic injection. Furthermore, there are reports available on the birth of fertile offspring following embryo transfer of such fertilized oocytes [125]. Thus, derivation of male germ cells from iPSCs epitomized mechanisms underlying the production of male gametes and their development, and also iPSCs have shown a ray of hope for confronting with infertility [127].

### iPSCs in veterinary science

Among farm animals, pig is considered a valuable model for testing drugs and therapeutics before they are introduced to clinics [127]. Therefore, utilization of large animal models has a great potential in this field. Recently, mesenchymal stem cells derived from equine iPSCs were used as a model for pre-clinical validation of stem cell therapies for muscles, joints, tendons, ligaments, and bone injuries [128]. The iPS technology offers potential applications in veterinary sciences other than therapeutics and biomedical. This technique could be employed in assisted reproduction technology for increasing cloning efficiency using iPSCs as potent nucleus donors instead of somatic cells or ESCs [23,129]. These reprogrammed cells could boost up production of cloned animals and hence helps in conservation of endangered species. iPSCs also help in generation of chimeric and transgenic animals [130-133].

## Conclusion

iPSCs have emerged as an alternative to stem cells in the field of biomedical sciences and research. Since iPSCs can omit the ethical concerns related to ESC derivation and potential issues of allogeneic rejection; therefore, the quintessential role of iPSCs leads to its use in therapy, drug development and clinical applications. The iPSCs hold great promise in the field of cell replacement therapy of diseases such as Alzheimer’s disease, Parkinson’s disease, cardiovascular disease, diabetes, and ALS. The iPS technology is a state-of-the-art technique in the field of sciences which holds copious thought-provoking applications in biomedical research. For the maximal and pertinent application of this technique in clinical purposes, we require a more widespread knowledge about the pros and cons of the reprogramming process and hope for a better future.

## Authors’ Contributions

NR and MKS contributed the relevant literature in preparation of this review and approved the final manuscript.

## References

[1] Martello G, Smith A (2014). Nature of embryonic stem cells annual review of cell and developmental biology. Cell. Dev. Biol.

[2] Bernard L, Lindsay P (2009). Ethical issues in stem cell research. Endocr. Rev.

[3] Wilmut I, Schnieke A.E, Mcwhir J, Kind A.J, Campbell K.H.S (1997). Viable offspring derived from fetal and adult mammalian cells. Nature.

[4] Silva J, Chambers I, Pollard S, Smith A (2006). Nanog promotes transfer of pluripotency after cell fusion. Nature.

[5] Pralong D, Trounson A.O, Verma P.J (2006). Cell fusion for reprogramming pluripotency:Toward elimination of the pluripotent genome. Stem Cell Rev.

[6] Yamanaka S, Blau H.M (2010). Nuclear reprogramming to a pluripotent state by three approaches. Nature.

[7] Bilic J, Carlos J, Belmonte I (2011). 2011 review:Induced pluripotent stem cells versus embryonic stem cells:Close enough or yet too far apart?. Stem Cells.

[8] Joo J.Y, Choi H.W, Kim M.J, Zaehres H, Tapia N, Stehling M, Jung K.S, Do J.T, Scholer H.R (2014). Establishment of a primed pluripotent epiblast stem cell in FGF4-based conditions. Sci. Rep.

[9] Weinberger L, Ayyash M, Novershtern N, Hanna J.H (2016). Dynamic stem cell states:Naive to primed pluripotency in rodents and humans. Nat. Rev. Mol. Cell. Biol.

[10] Li X, Pei D, Zheng H (2014). Transitions between epithelial and mesenchymal states during cell fate conversions. Prote. Cell.

[11] Hawkins K, Joy S, McKay T (2014). Cell signalling pathways underlying induced pluripotent stem cell reprogramming. World J. Stem Cells.

[12] Maherali N, Hochedlinger K (2009). TGF beta signal inhibition cooperates in the induction of iPSCs and replaces Sox2 and cMyc. Curr Biol.

[13] Jiao J, Dang Y, Yang Y, Gao R, Zhang Y, Kou Z, Sun X.F, Gao S (2013). Promoting reprogramming by FGF2 reveals that the extracellular matrix is a barrier for reprogramming fibroblasts to pluripotency. Stem Cells.

[14] Chambers I, Tomlinson S.R (2009). The transcriptional foundation of pluripotency. Development.

[15] Niwa H, Miyazaki J, Smith A.G (2000). Quantitative expression of Oct-3/4 defines differentiation, dedifferentiation or self-renewal of ES cells. Nat. Genet.

[16] Takahashi K, Yamanaka S (2006). Induction of pluripotent stem cells from mouse embryonic and adult fibroblast cultures by defined factors. Cell.

[17] Okita K, Ichisaka T, Yamanaka S (2007). Generation of germline-competent induced pluripotent stem cells. Nature.

[18] Wernig M (2007). *In vitro* reprogrammed fibroblasts have a similar developmental potential as ES cells and an ES cell-like epigenetic state. Nature.

[19] Du D, Lou X (2014). Generation of induced pluripotent stem cells from neonatal mouse cochlear cells. Differentiation.

[20] Koh S, Piedrahita J.A (2015). Generation of induced pluripotent stem cells (iPSCs) from adult canine fibroblasts methods. Mol. Biol.

[21] Esteban M.A, Xu J, Yang J, Peng M, Qin D, Li W, Jiang Z, Chen J, Deng K, Zhong M, Cai J, Lai L, Pei D (2009). Generation of induced pluripotent stem cell lines from Tibetan miniature pig. J. Biol. Chem.

[22] Fujishiro S.H, Nakano K, Mizukami Y, Azami T, Arai Y, Matsunari H, Ishino R, Nishimura T, Watanabe M, Abe T, Furukawa Y, Umeyama K, Yamanaka S, Ema M, Nagashima H, Hanazono Y (2013). Generation of naive-like porcine-induced pluripotent stem cells capable of contributing to embryonic and fetal development. Stem Cells Dev.

[23] Han X, Han J, Ding F, Cao S, Lim S.S, Dai Y, Zhang R, Zhang Y, Lim B, Li N (2011). Generation of induced pluripotent stem cells from bovine embryonic fibroblast cells. Cell Res.

[24] Deng Y, Liu Q, Luo C, Chen S, Li X, Wang C, Liu Z, Lei X, Zhang H, Sun H, Lu F, Jiang J, Shi D (2012). Generation of induced pluripotent stem cells from buffalo (*Bubalus bubalis*) fetal fibroblasts with buffalo defined factors. Stem Cells Dev.

[25] Imamura M, Okuno H, Tomioka I, Kawamura Y, Lin Z.Y, Nakajima R, Akamatsu W, Okano J, Matsuzaki Y, Sasaki E, Okano H (2012). Derivation of induced pluripotent stem cells by retroviral gene transduction in mammalian species. Methods Mol. Biol.

[26] Takahashi K, Tanabe K, Ohnuki M, Narita M, Ichisaka T, Tomoda K, Yamanaka S (2007). Induction of pluripotent stem cells from adult human fibroblasts by defined factors. Cell.

[27] Ninagawa N.T, Kawabata Y, Watanabe S, Nagata K, Torihashi S (2014). Generation of rat-induced pluripotent stem cells from a new model of metabolic syndrome. Plos One.

[28] Honda A, Hatori M, Hirose M, Honda C, Izu H, Inoue K, Hirasawa R, Matoba S, Togayachi S, Hiroyuki M, Atsuo O (2013). Naïve-like conversion overcomes the limited differentiation capacity of induced pluripotent stem cells. J. Biol. Chem.

[29] Sandmaier S.E, Nandal A, Powell A, Garrett W, Blomberg L, Donovan D.M, Talbot N, Telugu B.P (2015). Generation of induced pluripotent stem cells from domestic goats. Mol. Reprod. Dev.

[30] Cao H, Yang P, Pu Y, Sun X, Yin H, Zhang Y, Zhang Y, Li Y, Liu Y, Fang F, Zhang Z, Tao Y, Zhang X (2012). Characterization of bovine induced pluripotent stem cells by lentiviral transduction of reprogramming factor fusion proteins. Int. J. Biol. Sci.

[31] Wang J, Gu Q, Hao J, Bai D, Liu L, Zhao X, Liu Z, Wang L, Zhou Q (2013). Generation of induced pluripotent stem cells with high efficiency from human umbilical cord blood mononuclear cells. Dev. Reprod. Biol.

[32] Sommer C.A, Sommer A.G, Longmire T.A, Christodoulou C, Thomas D.D, Gostissa M, Alt F.W, Murphy G.J, Kotton D.N, Mostoslavsky G (2010). Excision of reprogramming transgenes improves the differentiation potential of iPS cells generated with a single excisable vector. Stem Cells.

[33] Meng F, Wang X, Gu P, Wang Z, Guo W (2013). Induction of retinal ganglion-like cells from fibroblasts by adenoviral gene delivery. Neuroscience.

[34] Fujie Y, Fusaki N, Katayama T, Hamasaki M, Soejima Y, Soga M, Ban H, Hasegawa M, Yamashita S, Kimura S, Suzuki S, Matsuzawa T, Akari H, Takumi E (2014). New type of *Sendai virus* vector provides transgene-free iPS cells derived from chimpanzee blood. PLoS One.

[35] Kishino Y, Seki T, Yuasa S, Fujita J, Fukuda K (2015). Generation of induced pluripotent stem cells from human peripheral T cells using *Sendai virus* in feeder-free conditions. J. Vis. Exp.

[36] Xue Y, Cai X, Wang L, Liao B, Zhang H, Shan Y, Chen Q, Zhou T, Li X, Hou J, Chen S, Luo R, Qin D, Pei D, Pan G (2013). Generating a non-integrating human induced pluripotent stem cell bank from urine-derived cells. PLoS One.

[37] Wen W, Zhang J, Xu J, Su R.J, Neises A, Ji G.Z, Yuan W, Cheng T, Zhang X.B (2016). Enhanced generation of integration-free iPSCs from human adult peripheral blood mononuclear cells with an optimal combination of episomal vectors. Stem Cell Rep.

[38] Meraviglia V, Zanon A, Lavdas A.A, Schwienbacher C, Silipigni R, Segni M, Chen H.S, Pramstaller P.P, Hicks A.A, Rossini A (2015). Generation of induced pluripotent stem cells from frozen buffy coats using non-integrating episomal plasmids. J. Vis. Exp.

[39] Talluri T.R, Kumar D, Glage S, Garrels W, Ivics Z, Debowski K, Behr R, Niemann H, Kues W.A (2015). Derivation and characterization of bovine induced pluripotent stem cells by transposon-mediated reprogramming. Cell Reprogram.

[40] Mo X, Li N, Wu S (2014). Generation and characterization of bat-induced pluripotent stem cells. Theriogenology.

[41] Choi H.Y, Lee T.J, Yang G.M, Oh J, Won J, Han J, Jeong G.J, Kim J, Kim J.H, Kim B.S, Cho S.G (2016). Efficient mRNA delivery with graphene oxide-polyethylenimine for generation of footprint-free human induced pluripotent stem cells. J. Controll Release.

[42] Preskey D, Allison T.F, Jones M, Mamchaoui K, Unger C (2016). Synthetically modified mRNA for efficient and fast human iPS cell generation and direct trans differentiation to myoblasts. Biochem. Biophys. Res. Commun.

[43] Yoshioka N, Gros E, Li R, Kumar S, Deacon D.C, Maron C, Muotri A.R, Chi N.C, Fu X.D, Yu B.D, Dowdy S.F (2013). Efficient generation of human iPS cells by a synthetic self replicative RNA. Cell Stem Cell.

[44] Nemes C, Varga E, Polgar Z, Klincumhom N, Pirity M.K, Dinnyes A (2014). Generation of mouse induced pluripotent stem cells by protein transduction. Tissue Eng. Methods.

[45] Khan M, Narayana K, Lu H, Choo Y, Du C, Wiradharma N, Yang Y.Y, Wan A.C (2013). Delivery of reprogramming factors into fibroblasts for generation of non-genetic induced pluripotent stem cells using a cationic bolaamphiphile as a non-viral vector. Biomaterials.

[46] Cho S.J, Choi H.W, Cho J, Jung S, Seo H.G, Do J.T (2013). Activation of pluripotency genes by a nanotube-mediated protein delivery system. Mol. Reprod.

[47] Hirai H, Firpo M, Kikyo N (2012). Establishment of LIF-dependent human iPS cells closely related to basic FGF-dependent authentic iPS cells. PLoS One.

[48] Mohyeldin A, Garzon M.T, Quinones H.A (2010). Oxygen in stem cell biology:A critical component of the stem cell niche. Cell Stem Cell.

[49] Zhang Z, Wu W.S (2013). Sodium butyrate promotes generation of human induced pluripotent stem cells through induction of the miR302/367 cluster. Stem Cells Dev.

[50] Hou P, Li Y, Zhang X, Liu C, Guan J, Li H, Zhao T, Ye J, Yang W, Liu K (2013). Pluripotent stem cells induced from mouse somatic cells by small-molecule compounds. Science.

[51] Wu Y.L, Pandian G.N, Ding Y.P, Zhang W, Tanaka Y, Sugiyama H (2013). Clinical grade iPS cells:Need for versatile small molecules and optimal cell sources. Chem. Biol.

[52] Ichida J.K, Blanchard J, Lam K, Son E.Y, Chung J.E, Egli D, Loh K.M, Carter A.C, Giorgio K.K, Huangfu D, Akutsu H, Liu D.R, Rubin L.L, Eggan K (2009). A small molecule inhibitor of TGF-βsignaling replaces Sox2 in reprogramming by inducing nanog. Cell Stem Cell.

[53] Wang Y, Liang P, Lan F, Wu H, Lisowski L, Gu M, Hu S, Kay M.A, Urnov F.D, Shinnawi R (2014). Genome editing of isogenic human induced pluripotent stem cells recapitulates long QT phenotype for drug testing. J. Am. Coll. Cardiol.

[54] Li D, Wang L, Hou J, Shen Q, Chen Q, Wang X, Du J, Cai X, Shan Y, Zhang T, Zhou T, Shi X, Li Y, Zhang H, Guangjin P (2016). Optimized approaches for generation of integration-free iPSCs from human urine-derived cells with small molecules and autologous feeder. Stem Cell Rep.

[55] Lin T, Ambasudhan R, Yuan X, Li W, Hilcove S, Abujarour R, Lin X, Hahm H.S, Hao E, Hayek A (2009). A chemical platform for improved induction of human iPSCs. Nat. Methods.

[56] Shi Y, Desponts C, Do J.T, Hahm H.S, Scholer H.R, Ding S (2008). Induction of pluripotent stem cells from mouse embryonic fibroblasts by October 4 and Klf4 with small-molecule compounds. Cell Stem Cell.

[57] Pandian G.N, Sato S, Anandha K, Taniguchi J, Takashima K, Syed J, Han L, Saha A, Bando T, Nagase H (2014). Identification of a small molecule that turns on the pluripotency gene circuitry in human fibroblasts. ACS Chem. Biol.

[58] Debeb B.G, Lacerda L, Xu W, Larson R, Solley T, Atkinson R, Sulman E.P, Ueno N.T, Krishnamurthy S, Reuben J.M, Buchholz T.A, Woodward W.A (2012). Histone deacetylase inhibitors stimulate dedifferentiation of human breast cancer cells through WNT/β-catenin signalling. Stem Cells.

[59] Zhai Y, Chen X, Yu D, Li T, Cui J, Wang G, Hu J.F, Li W (2015). Histone deacetylase inhibitor valproic acid promotes the induction of pluripotency in mouse fibroblasts by suppressing reprogramming-induced senescence stress. Exp. Cell Res.

[60] Vazquez M.A, Corominas F.B, Cufi S, Vellon L, Oliveras F.C, Menendez O.J, Joven J, Lupu R, Menendez J.A (2013). The mitochondrial H(+)-ATP synthase and the lipogenic switch:New core components of metabolic reprogramming in induced pluripotent stem (iPS) cells. Cell Cycle.

[61] Bernhardt M, Galach M, Novak D, Utikal J (2012). Mediators of induced pluripotency and their role in cancer cells-current scientific knowledge and future perspectives. Biotechnol. J.

[62] Lee D.F, Su J, Kim H.S, Chang B, Papatsenko D, Zhao R, Yuan Y, Gingold J, Xia W, Darr H, Mirzayans R, Hung M.C, Schanie C, Ihor R, Lemischka I.R (2015). Modeling familial cancer with induced pluripotent stem cells. Cell.

[63] Berdasco M, Esteller M (2010). Aberrant epigenetic landscape in cancer:How cellular identity goes awry. Dev. Cell.

[64] Lin S.L, Chang D.C, Chang L.S (2008). 2008-302 reprograms human skin cancer cells into a pluripotent ES-cell-like state. RNA.

[65] Utikal J, Maherali N, Kulalert W, Hochedlinger K (2009). Sox2 is dispensable for the reprogramming of melanocytes and melanoma cells into induced pluripotent stem cells. J. Cell Sci.

[66] Miyoshi N, Ishii H, Nagai K (2010). Defined factors induce reprogramming of gastrointestinal cancer cells. Proc. Natl. Acad. Sci.

[67] Carette J.E, Pruszak J, Varadarajan M (2010). Generation of iPSCs from cultured human malignant cells. Blood.

[68] Mathieu J, Zhang Z, Zhou W (2011). HIF induces human embryonic stem cell markers in cancer cells. Cancer Res.

[69] Corominas F.B, Cufi S, Oliveras F.C (2013). Nuclear reprogramming of luminal-like breast cancer cells generates Sox2-overexpressing cancer stem-like cellular states harboring transcriptional activation of the mTOR pathway. Cell Cycle.

[70] Stricker S.H, Feber A, Engstrom P.G (2013). Widespread resetting of DNA methylation in glioblastoma-initiating cells suppresses malignant cellular behaviour in a lineage-dependent manner. Genes Dev.

[71] Zhang J (2011). A human iPSC model of hutchinson gilford progeria reveals vascular smooth muscle and mesenchymal stem cell defects. Cell Stem Cell.

[72] Broxmeyer H.E (2010). 2010 iPS cells enhance therapeutic applicability of cord blood cells and banking?. Cell Stem Cell.

[73] Hanna J, Wernig M, Markoulaki S, Sun C.W, Meissner A, Cassady J.P, Beard C, Brambrink T, Wu L.C, Townes T.M, Jaenisch R (2007). Treatment of sickle cell anemia mouse model with iPS cells generated from autologous skin. Science.

[74] Tsuji O, Miura K, Okada Y, Fujiyoshi K, Mukaino M, Nagoshi N, Kitamura K, Kumagai G, Nishino M, Tomisato S (2010). Therapeutic potential of appropriately evaluated safe induced pluripotent stem cells for spinal cord injury. Proc. Natl. Acad. Sci.

[75] Wernig M, Zhao J.P, Pruszak J, Hedlund E, Fu D, Soldner F, Broccoli V, Paton M, Isacson O, Jaenisch R (2008). Neurons derived from reprogrammed fibroblasts functionally integrate into the fetal brain and improve symptoms of rats with Parkinson’s disease. Proc. Natl. Acad. Sci.

[76] Swistowski A, Peng J, Liu Q, Mali P, Rao M.S, Cheng L, Zeng X (2010). Efficient generation of functional dopaminergic neurons from human induced pluripotent stem cells under defined conditions. Stem Cells.

[77] Xu D, Alipio Z, Fink L.M, Adcock D.M, Yang J, Ward D.C, Ma Y (2009). Phenotypic correction of murine hemophilia A using an iPS cell-based therapy. Proc. Natl. Acad. Sci.

[78] Kang S, Xia J.C, Lai W (2010). Functional mesenchymal stem cells derived from human induced pluripotent stem cells attenuate limb ischemia in mice. Circulation.

[79] Nelson T.J, Martinez F.A, Yamada S, Perez T.C, Ikeda Y, Terzic A (2009). Repair of acute myocardial infarction by human stemness factors induced pluripotent stem cells. Circulation.

[80] Funakoshi S, Miki K, Takaki T, Okubo C, Hatani T, Chonabayashi K, Nishikawa M, Takei I, Oishi A, Narita M, Hoshijima M, Kimura T, Yamanaka S, Yoshida Y (2016). Enhanced engraftment, proliferation, and therapeutic potential in heart using optimized human iPSC-derived cardiomyocytes. Sci. Rep.

[81] Rufaihah A.J, Huang N.F, Jame S, Lee J.C, Nguyen H.N, Byers B, De A, Okogbaa J, Rollins M, Reijo R (2011). Endothelial cells derived from human iPSCS increase capillary density and improve perfusion in a mouse model of peripheral arterial disease. Arterioscler Thromb. Vasc. Biol.

[82] Alipio Z, Liao W, Roemer E.J, Waner M, Fink L.M, Ward D.C, Ma Y (2010). Reversal of hyperglycemia in diabetic mouse models using induced-pluripotent stem (iPS)-derived pancreatic β-like cells. Proc. Natl. Acad. Sci.

[83] Liu H, Kim Y, Sharkis S, Marchionni L, Jang Y.Y (2011). *In vivo* liver regeneration potential of human induced pluripotent stem cells from diverse origins. Sci. Transl. Med.

[84] Garber K (2015). RIKEN suspends first clinical trial involving induced pluripotent stem cells. Nat. Biotechnol.

[85] Urbach A (2010). Differential modeling of fragile X syndrome by human embryonic stem cells and induced pluripotent stem cells. Cell Stem Cell.

[86] Tolar J, Park I.H, Xia L, Lees C.J, Peacock B, Webber B, McElmurry R.T, Eide C.R, Orchard P.J, Kyba M, Osborn M.J, Lund T.C, Wagner J.E, Daley G.Q, Blazar B.R (2011). Hematopoietic differentiation of induced pluripotent stem cells from patients with mucopolysaccharidosis type I (Hurler syndrome). Blood.

[87] Liu H (2010). Generation of endoderm derived human induced pluripotent stem cells from primary hepatocytes. Hepatology.

[88] Sullivan G.J (2010). Generation of functional human hepatic endoderm from human induced pluripotent stem cells. Hepatology.

[89] Li Z, Hu S, Ghosh Z, Han Z, Wu J.C (2011). Functional characterization and expression profiling of human induced pluripotent stem cell and embryonic stem cell derived endothelial cells. Stem Cells Dev.

[90] Dimos J.T (2008). Induced pluripotent stem cells generated from patients with ALS can be differentiated into motor neurons. Science.

[91] Ebert A.D, Yu J, Rose F.F, Mattis V.B, Lorson C.L, Thomson J.A, Svendsen C.N (2009). Induced pluripotent stem cells from a spinal muscular atrophy patient. Nature.

[92] Marchetto M.C (2010). A model for neural development and treatment of Rett syndrome using human induced pluripotent stem cells. Cell.

[93] Chamberlain S.J (2010). Induced pluripotent stem cell models of the genomic imprinting disorders Angelman and Prader Willi syndromes. Proc. Natl. Acad. Sci.

[94] Narsinh K, Narsinh K.H, Wu J.C (2011). Derivation of human induced pluripotent stem cells for cardiovascular disease modelling. Circ. Res.

[95] Yazawa M, Hsueh B, Jia X, Pasca A.M, Bernstein J.A, Hallmayer J, Dolmetsch R.E (2011). Using induced pluripotent stem cells to investigate cardiac phenotypes in Timothy syndrome. Nature.

[96] Ebert A.D, Liang P, Wu J.C (2012). 2012 pluripotent stem cells as a disease modelling and drug screening platform. J Cardiovasc. Pharmacol.

[97] Lan F, Lee A.S, Liang P, Sanchez V, Nguyen P.K, Wang L, Han L, Yen M, Wang Y, Sun N (2013). Abnormal calcium handling properties underlie familial hypertrophic cardiomyopathy pathology in patient-specific induced pluripotent stem cells. Cell Stem Cell.

[98] Ye L, Chang J.C, Lin C, Sun X, Yu J, Kan Y.W (2009). Induced pluripotent stem cells offer new approach to therapy in thalassemia and sickle cell anemia and option in prenatal diagnosis in genetic diseases. Proc. Natl. Acad. Sci.

[99] Soldner F, Hockemeyer D, Beard C, Gao Q, Bell G.W, Cook E.G, Hargus G, Blak A, Cooper O, Mitalipova M (2009). Parkinson’s disease patient-derived induced pluripotent stem cells free of viral reprogramming factors. Cell.

[100] Kang J, Tang B, Guo J (2016). The progress of induced pluripotent stem cells as models of Parkinson’s disease. Stem Cells Int.

[101] Sato Y, Kobayashi H, Higuchi T, Shimada Y, Era T, Kimura S, Eto Y, Ida H, Ohashi T (2015). Disease modelling and lentiviral gene transfer in patient specific induced pluripotent stem cells from late onset Pompe disease patient. Mol. Ther. Methods Clin. Dev.

[102] Hotta A, Cheung A.Y, Farra N, Vijayaragavan K, Séguin C.A, Draper J.S, Pasceri P, Maksakova I.A, Mager D.L, Rossant J, Bhatia M, Ellis J (2009). Isolation of human iPS cells using EOS lentiviral vectors to select for pluripotency. Nat. Methods.

[103] Park I.H, Arora N, Huo H, Maherali N, Ahfeldt T, Shimamura A, Lensch M.W, Cowan C, Hochedlinger K, Daley G.Q (2008). Disease-specific induced pluripotent stem (iPS) cells. Cell.

[104] Carvajal V.X, Sevilla A, D’Souza S.L, Ang Y.S, Schaniel C, Lee D.F, Yang L, Kaplan A.D, Adler E.D, Rozov R (2010). Patient-specific induced pluripotent stem-cell-derived models of LEOPARD syndrome. Nature.

[105] Ebert A.D, Kodo K, Liang P, Wu H, Huber B.C, Riegler J, Churko J, Lee J, Almeida P, Lan F (2014). Characterization of the molecular mechanisms underlying increased ischemic damage in the aldehyde dehydrogenase 2 genetic polymorphism using a human induced pluripotent stem cell model system. Sci. Transl. Med.

[106] Ebert A.D, Diecke S, Chen I.Y, Wu J.C (2015). 2015 and transdifferentiation for cardiovascular development and regenerative medicine:Where do we stand?. EMBO Mol. Med.

[107] Kim C, Wong J, Wen J, Wang S, Wang C, Spiering S, Kan N.G, Forcales S, Puri P.L, Leone T.C (2013). Studying arrhythmogenic right ventricular dysplasia with patient-specific iPSCs. Nature.

[108] Asimaki A, Kapoor S, Plovie E, Karin A.A, Adams E, Liu Z, James C.A, Judge D.P, Calkins H, Churko J (2014). Identification of a new modulator of the intercalated disc in a zebrafish model of arrhythmogenic cardiomyopathy. Sci. Transl. Med.

[109] Moretti A, Bellin M, Welling A, Jung C.B, Lam J.T, Bott F.L, Dorn T, Goedel A, Hohnke C, Hofmann F (2010). Patient-specific induced pluripotent stem-cell models for long-QT syndrome. N. Engl. J. Med.

[110] Itzhaki I, Maizels L, Huber I, Dantsis L, Caspi O, Winterstern A, Feldman O, Gepstein A, Arbel G, Hammerman H (2011). Modelling the long QT syndrome with induced pluripotent stem cells. Nature.

[111] Sun N, Yazawa M, Liu J, Han L, Sanchez F.V, Abilez O.J, Navarrete E.G, Hu S, Wang L, Lee A (2012). Patient-specific induced pluripotent stem cells as a model for familial dilated cardiomyopathy. Sci. Transl. Med.

[112] Sharma A, Marceau C, Hamaguchi R, Burridge P.W, Rajarajan K, Churko J.M, Wu H, Sallam K.I, Matsa E, Sturzu A.C (2014). Human induced pluripotent stem cell-derived cardiomyocytes as an *in vitro* model for coxsackievirus B3-induced myocarditis and antiviral drug screening platform. Circ. Res.

[113] Dick E (2010). Evaluating the utility of cardiomyocytes from human pluripotent stem cells for drug screening. Biochem. Soc. Trans.

[114] Zhao J, Jiang W.J, Chen S, Hou C.Z, Yang X.M, Gao J.G (2013). Induced pluripotent stem cells:Origins, applications, and future perspectives. Biomed. Biotechnol.

[115] Lee G, Papapetrou E.P, Kim H, Chambers S.M, Tomishima M.J, Fasano C.A, Ganat Y.M, Menon J, Shimizu F, Viale A (2009). Modelling pathogenesis and treatment of familial dysautonomia using patient specific iPS cells. Nature.

[116] Drawnel F.M, Boccardo S, Prummer M, Delobel F, Graff A, Weber M, Gérard R, Thong L.B, Bu L, Jiang X, Hoflack J.C, Kiialainen A, Jeworutzki E, Aoyama N, Carlson C, Burcin M, Gromo G, Boehringer M, Stahlberg H, Hall B.J, Magnone M.C, Kolaja K, Chien K.R, Bailly J, Iacone R (2014). Disease modelling and phenotypic drug screening for diabetic cardiomyopathy using human induced pluripotent. Stem Cell Rep.

[117] Xu X.H, Zhong Z (2013). Disease modelling and drug screening for neurological diseases using human induced pluripotent stem cells. Acta Pharmacol. Sin.

[118] Egawa N, Kitaoka S, Tsukita K, Naitoh M, Takahashi K, Yamamoto T, Adachi F, Kondo T, Okita K, Asaka I, Aoi T, Watanabe A, Yamada Y, Morizane A, Takahashi J, Ayaki T, Ito H, Yoshikawa K, Yamawaki S, Suzuki S, Watanabe D, Hioki D, Kaneko T, Makioka K, Okamoto K, Takuma H, Tamaoka A, Hasegawa K, Nonaka T, Hasegawa M, Kawata A, Yoshida M, Nakahata T, Takahashi R, Marchetto M.C, Gage F.H, Yamanaka S, Inoue H (2012). Drug screening for ALS using patient-specific induced pluripotent stem cells. Sci. Transl. Med.

[119] Davidson M.D, Ware B.R, Khetani S.R (2015). Stem cell-derived liver cells for drug testing and disease modelling. Discov. Med.

[120] Zhu Y, Hu L, Li P (2012). Generation of male germ cells from induced pluripotent stem cells (iPS cells):An *in vitro* and *in vivo* study. Asian J. Androl.

[121] Yang S, Bo J, Hu H (2012). Derivation of male germ cells from induced pluripotent stem cells *in vitro* and in reconstituted seminiferous tubules. Cell Prolif.

[122] Li P, Hu H, Yang S (2013). Differentiation of induced pluripotent stem cells into male germ cells *in vitro* through embryoid body formation and retinoic acid or testosterone induction. Bio Med. Res. Int.

[123] Malik N, Rao M.S (2013). A review of the methods for human iPSC derivation. Methods Mol. Biol.

[124] Xu X, Yi F.Z, Pan H (2013). Progress and prospects in stem cell therapy. Acta Pharmacol. Sin.

[125] Hayashi K, Ohta H, Kurimoto K, Aramaki S, Saitou M (2011). Reconstitution of the mouse germ cell specification pathway in culture by pluripotent stem cells. Cell.

[126] Ohinata Y, Ohta H, Shigeta M, Yamanaka K, Wakayama T, Saitou M (2009). A signaling principle for the specification of the germ cell lineage in mice. Cell.

[127] Volarevic V, Bojic S, Nurkovic J, Volarevic A, Ljujic B, Arsenijevic N, Lako M, Stojkovic M (2014). Stem cells as new agents for the treatment of infertility:Current and future perspectives and challenges. Bio Med. Res. Int.

[128] Brevini T.A, Antonini S, Cillo F, Crestan M, Gandolfi F (2007). Porcine embryonic stem cells:Facts, challenges and hopes. Theriogenology.

[129] Nagy K, Sung H.K, Zhang P, Laflamme S, Vincent P, Mohammadi S (2011). Induced pluripotent stem cell lines derived from equine fibroblasts. Stem Cell Rev.

[130] Fan N, Chen J, Shang Z, Dou H, Ji G, Zou Q (2013). Piglets cloned from induced pluripotent stem cells. Cell Res.

[131] West F.D, Terlouw S.L, Kwon D.J, Mumaw J.L, Dhara S.K, Hasneen K, Dobrinsky J.R, Stice S.L (2010). Porcine induced pluripotent stem cells produce chimeric off springs. Stem Cells Dev.

[132] Baker M (2009). iPS cells make mice that make mice. Nat. Rep. Stem Cells.

[133] Guo J, Baojiang W, Shuyu L, Siqin B, Lixia Z, Shuxiang H, Wei S, Jie S, Yangfeng D, Xihe L (2014). Contribution of mouse embryonic stem cells and induced pluripotent stem cells to chimeras through injection and co-culture of embryos. Stem Cell Int.

